# Behaviour of aqueous sulfamethizole solution and temperature effects in cold plasma oxidation treatment

**DOI:** 10.1038/s41598-018-27061-5

**Published:** 2018-06-07

**Authors:** Alexander Sokolov, Marjatta Louhi-Kultanen

**Affiliations:** 1 School of Engineering Science, Lappeenranta University of Technology, P.O. Box 20, FI-53850 Lappeenranta, Finland; 2 School of Chemical Engineering, Aalto University, P.O. Box 16100, FI-00076 Aalto, Espoo Finland

## Abstract

The increase in volume and variety of pharmaceuticals found in natural water bodies has become an increasingly serious environmental problem. The implementation of cold plasma technology, specifically gas-phase pulsed corona discharge (PCD), for sulfamethizole abatement was studied in the present work. It was observed that sulfamethizole is easily oxidized by PCD. The flow rate and pH of the solution have no significant effect on the oxidation. Treatment at low pulse repetition frequency is preferable from the energy efficiency point of view but is more time-consuming. The maximum energy efficiency was around 120 g/kWh at half-life and around 50 g/kWh at the end of the treatment. Increasing the solution temperature from room temperature to 50 °C led to a significant reaction retardation of the process and decrease in energy efficiency. The pseudo-first order reaction rate constant (*k*_1_) grows with increase in pulse repetition frequency and does not depend on pH. By contrast, decreasing frequency leads to a reduction of the second order reaction rate constant (*k*_2_). At elevated temperature of 50 °C, the *k*_1_, *k*_2_ values decrease 2 and 2.9 times at 50 pps and 500 pps respectively. Lower temperature of 10 °C had no effect on oxidation efficiency compared with room temperature.

## Introduction

Despite pharmaceuticals having first been detected in natural water bodies more than 40 years ago, these compounds were, until recently, not considered hazardous as their concentrations were very low. To date, there is a trend of increasing pharmaceuticals concentrations in lakes, rivers and seas, due to a general increase in usage of pharmaceutical compounds for both medicinal purposes and in livestock production. Through the use of advanced analytical techniques, pharmaceuticals in extremely low concentrations have been detected in tap water^1,2^. The continuous increase in the prevalence of these compounds in natural water bodies is also due to an absence of legislation specifically addressing the discharge of pharmaceuticals-containing wastewaters into ground water and surface waters^3^. Existing municipal wastewater treatment plants are not designed for efficient removal of medical drugs from water, especially refractory compounds such as antibiotics^4–6^.

Over recent years, the global consumption of antibiotics has increased rapidly. According to Van Boeckel^7^, global consumption of antibiotics in the first decade of the 21^st^ century increased by 36%. One of the classes of antibiotics with greatest use is compounds belonging to the sulfonamide functional group. In Europe, total consumption of sulfonamides for human medicine was 121.5 tonnes of active pharmaceutical ingredient in 2012, which places the compound as the fifth most commonly used antimicrobial antibiotic. According to the European Centre for Disease Prevention and Control, Finland is among the largest per capita consumers of sulfonamides in Europe^8^. As regards consumption of veterinary antibiotics (mainly in livestock production), sulfonamides are the third most commonly used antibiotic in Europe with consumption of 826.3 tonnes of active ingredient in 2012.

Sulfamethizole is a typical representative of the sulfonamides group. It is quite popular in livestock farming, which poses problems as effluents from farms often go directly into water bodies bypassing wastewater treatment facilities^4,9^. Furthermore, according to Scholar and Pratt^10^, about 80% of the original intake of sulfamethizole is excreted. Other sources of antibiotic release into the environment are leaching from landfills^11^, recycled water utilized for groundwater recharge and irrigation^12–14^, and disposal of unused and expired pharmaceuticals^15^. Sulfamethizole was chosen as the test compound in this study for the following reasons: it is among the most commonly used antibiotics and overall consumption is high; a large amount of the administered dose is excreted; and it is among the most often detected antibiotics in environmental waters and, most importantly, in tap water.

As mentioned earlier, conventional wastewater treatment methods are inadequate for effective abatement of pharmaceuticals in the environment. Implementation of activated carbon treatment, separation by membrane using reverse osmosis, micro- and nanofiltration could be effective methods, but the high maintenance and operational costs of these processes limit the use of such approaches^16^. Ozonation has been found to be a quite effective method for antibiotics removal in general^17,18^ and for sulfamethizole in particular^16^. However, the ozone dosages commonly implemented in water treatment are insufficient for mineralization. Incomplete mineralization leads to the formation of oxidation byproducts and these products can show greater toxicity than the parent compounds^19–21^. Furthermore, ozonation remains an expensive method for water purification^22,23^. In light of the drawbacks of alternative approaches, advanced oxidation processes (AOP) based on hydroxyl radical oxidation have attracted increasing research interest. Ikehata *et al*. review the most popular AOPs and present the advantages and disadvantages of the different approaches^24^. For sulfamethizole, the most studied AOPs are photocatalytic oxidation, oxidation via the Fenton reaction, and oxidation by Ferrate(VI)^25–27^. Cold plasma technology can be considered as a novel AOP technology. For the most part, cold plasma treatment, in the form of oxidation of various compounds, has been associated with electric discharge systems. The current work studies implementation of gas-phase pulsed corona discharge for the treatment of recalcitrant pharmaceuticals. This method allows the generation of short-living OH radicals and long-living ozone from water and oxygen. The generation of oxidants takes place *in situ* with low delivered energy, and with minimum production of heat in the working chamber of the PCD reactor.

According to Ikonen *et al*.^28^, in 2014 there were 154 large (>5000 users or >1000 m^3^ drinking water/day) EU-regulated waterworks in Finland; 41% of them used groundwater in their drinking water production, 19% used artificially recharged groundwater, and the remaining 40% used surface water sources. Drinking water treatment is strongly dependent on water temperature. Due to the northern location of Finland, the temperature of water in water bodies can vary from 0 °C to 20 °C. Wastewater disposal from industrial plant can reach temperatures of 40–50 °C^29^. PCD has already proved an efficient method for pharmaceutical oxidation^30–32^ but the effect of the water temperature on the PCD oxidation treatment has not been studied. The current research investigates the oxidation of aqueous solutions of sulfamethizole in a cold plasma field at different water temperatures. The main aim is to estimate the effect of temperature on the oxidation kinetics, oxidation by-products and energy efficiency of the process.

## Materials and Methods

Commercially available sulfamethizole, supplied by Sigma Aldrich, was used for the experiments. The purity of the test compound, based on the manufacturer’s specification exceeded 99%.

The concentration of sulfamethizole in the studied aqueous solutions was measured by high performance liquid chromatography (HPLC). A Kinetex 2.6 µm C18 100 A 150 × 4.60 mm column was used for analysis of the studied solutions. The column temperature was 35 °C, retention time was around 13 minutes, and the wavelength was 254 nm. The eluent included 1% acetic acid solution and methanol in a volumetric proportion of 85:15 respectively. Eluent flow rate was 0.6 ml/min. Sample injection volume was 20 µl. The accuracy of these measurements was ± 0.1 mg/L.

The qualitative analysis of the oxidation by-products was carried out with chromatography coupled to an ion trap mass spectrometer (Agilent 1100 series LC/MSD Trap) equipped with an electrospray ionization interface. The analysis was performed with full scan and auto MS/MS modes in both positive and negative polarity. The neutral samples were injected without prior sample treatment and the pH of the alkaline samples was adjusted to 3 with formic acid before injection. An XBridge C_18_ column (2.1 × 50 mm, 3 μm, Waters Corp.) was used for chromatographic separation. The eluent consisted of acetonitrile with 0.1% formic acid, and the flow rate was 0.4 ml/min. The gradient applied was: 0–1 min, 5% eluent; 1–25 min, 5–95% eluent; 25–26 min, 95% eluent; 26–27 min, 95–5% eluent; 27–35 min, 5% eluent. The injection volume was 30 μL. The LC tool was equipped with an adjustable wavelength UV detector set at 275 nm. The source was: gas temperature 350 °C, gas flow 8 L/min, capillary voltage of ± 3.5 kV, nebulizer 40 psi.

A quadrupole time-of-flight mass spectrometer (Bruker MicrOTOF) equipped with an electrospray ionization interface was used for further investigation of the intermediates. The chromatographic method was the same as for the ion trap spectrometry. An Agilent series 1200 LC was used for chromatographic separations.

**Figure 1:**
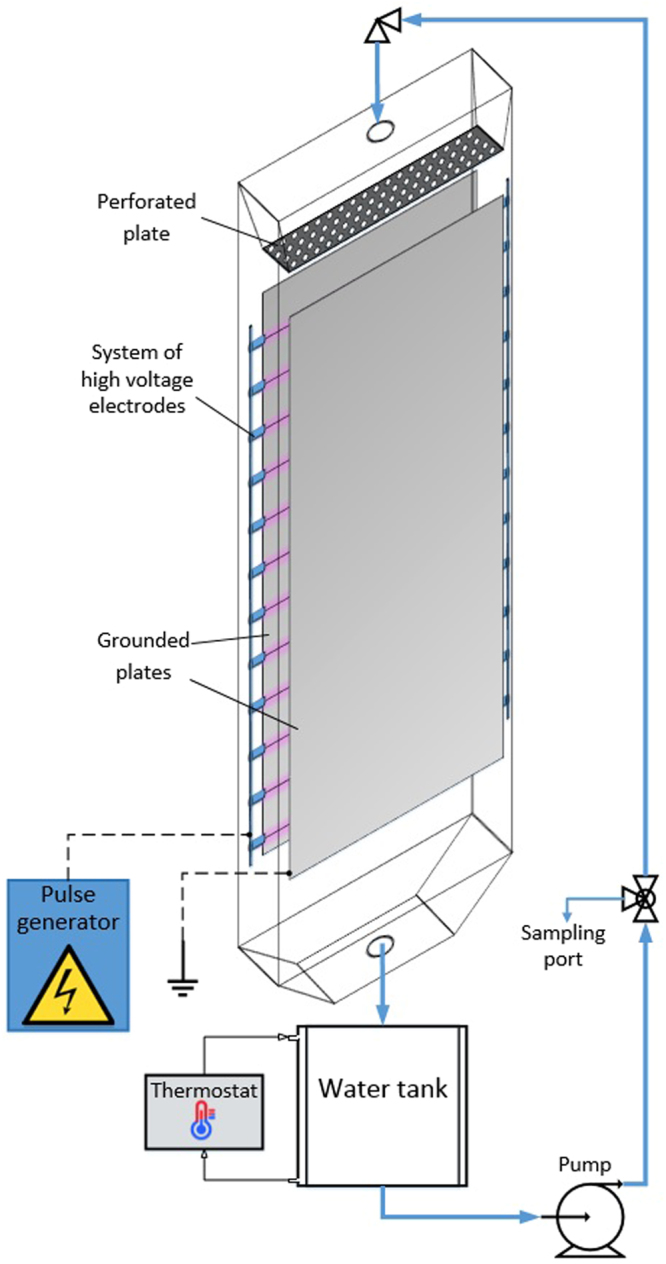
Experimental setup.

A schematic drawing of the experimental setup is shown on Fig. 1. The setup includes the PCD reactor, water circulation system, high voltage pulse generator and thermostat. The PCD reactor consists of two vertical grounded plate electrodes and a horizontal high voltage electrode wires between them. More detailed information on the reactor configuration is provided in our previous publication^32^. The sulfamethizole solution was pumped from the water tank to the top of the reactor, where it was spread by perforated plate and fell by gravity through the system of high voltage electrodes back to the water tank. The reaction between the target compound and oxidants takes place in the plasma zone between the grounded plates. In the plasma field, two main oxidants, ozone and hydroxyl radicals, are generated from water and oxygen via the oxidation reactions:

1 $${e}^{-}+{H}_{2}O\to {e}^{-}+\cdot OH$$

2 $${e}^{-}+3{O}_{2}\to {e}^{-}+2{O}_{3}$$

3 $$O+{H}_{2}O\to 2OH$$

To compare our results with other studies on the degradation of pharmaceutical compounds in water by electric discharge, target compound removal (R, %) and energy efficiency (ε, g/kWh,) were used as the main evaluation parameters. According to the literature review, these parameters are the most frequently used evaluation parameters in the field of degradation of various compounds by cold plasma treatment.

The compound removal and energy efficiency were calculated according to Equation (4) and Equation (5), respectively:

4 $$R=(1-{C}_{t}/{C}_{0})\times 100$$

5 $${\rm{\varepsilon }}={C}_{0}\,V\,R/(P\,t)$$

where C_0_ is the initial concentration of the target compound (mg/L) and C_t_ is the concentration at the time t (mg/L); t is the treatment time (h); V is the volume of treated solution (L); and P is the discharge power (W).

The value P depends on the pulse repetition frequency, which comprised 50, 200 and 500 pulses per second (pps), corresponding to P = 6 W, P = 24 W and P = 60 W, respectively. More detailed description of calculation of the P value is available in previous publications^22,32^.

All experiments were divided into three parts (see Table 1). The first part included experiments without power supply. The aqueous solution of sulfamethizole was pumped through the reactor for 5 hours at various temperatures of solution (20 °C and 50 °C) under neutral, alkaline and acidic conditions. Here and throughout the work, the term “neutral” refers to media without any additives (initial pH is around 7), the term “acidic” refers to media with sulfuric acid as an additive (initial pH is around 4), and the term “alkaline” refers to media with sodium hydroxide as an additive (initial pH is around 12). Samples were taken after each hour. This set of experiments was necessary to make sure that the sulfamethizole is stable and does not degrade by itself without treatment.

**Table 1:** List of experiments.

Frequency, pps	T, °C	initial pH (after treatment)	Total treatment time, min	*k*_1_, min^−1^	*k*_2_, m^3^/J	*t*_*1/2*_, min	*ε*_1/2_, g/kWh	*ε*_final_, g/kWh	Removal rate
**First set of experiments (without power supply)**									
0	20	7.1 (7.1)	300	—	—	—	—	—	—
0	20	12.2 (12.2)	300	—	—	—	—	—	—
0	20	3.5 (3.5)	300	—	—	—	—	—	—
0	50	7.2 (7.2)	300	—	—	—	—	—	—
0	50	12.0 (12.0)	300	—	—	—	—	—	—
0	50	3.5 (3.5)	300	—	—	—	—	—	—
Second set of experiments*									
50	20	7.1 (4.0)	100	0.03311	6.57 × 10^−7^	20.93	122.6	49.92	0.99
50	20	12.1 (11.9)	100	0.03289	6.52 × 10^−7^	21.07	120.9	49.17	0.99
50	20	3.5 (3.1)	100	0.03321	6.59 × 10^−7^	20.87	117.8	48.11	1.00
200	20	7.2 (4.0)	40	0.08760	4.34 × 10^−7^	7.91	81.5	30.89	1.00
200	20	12.2 (12.0)	40	0.09557	4.74 × 10^−7^	7.25	88.7	31.47	0.99
200	20	3.6 (3.1)	40	0.08701	4.31 × 10^−7^	7.97	79.4	30.45	1.00
500	20	7.2 (4.1)	24	0.15300	3.03 × 10^−7^	4.53	56.2	20.75	1.00
500	20	12.1 (12.1)	24	0.1616	3.21 × 10^−7^	4.29	60.2	21.00	0.99
500	20	3.5 (3.2)	24	0.1380	2.74 × 10^−7^	5.02	49.1	19.92	1.00
**Third set of experiments**									
50	50	7.0 (5.8)	160	0,01622	3.22 × 10^−7^	42.73	59.8	30.15 (20.5)**	0.97 (0.99)**
50	10	7.0 (4.7)	100	0,03425	6.79 × 10^−7^	20.24	127.4	49.38	0.99
500	50	6.9 (4.4)	60	0,05295	1.05 × 10^−7^	13.09	19.6	8.11 (6.2)**	0.97 (0.99)**
500	10	7.0 (4.9)	24	0,1594	3.16 × 10^−7^	4.35	58.4	20.84	1.00

^*^The same set was repeated with the flow rate 8 L/min.

**After approximation.

The second set of experiments were carried out at three different pulse repetition frequencies (50 pps, 200 pps and 500 pps) under initial neutral, alkaline and acidic conditions. The process was operated with two flow rates of circulating aqueous solution – 4.5 L/min and 8 L/min. Samples were taken with treatment time as indicated in Table 2. The second set was necessary to obtain information about the general behavior of sulfamethizole in a cold plasma field.

**Table 2:** Corresponding energy delivered with treatment time.

Delivered energy dose (kWh/m^3^)	Treatment time for 50 pps (min)	Treatment time for 200 pps (min)	Treatment time for 500 pps (min)
0	0	0	0
0.1	10	2.5	—
0.2	20	5	—
0.4	40	10	4
1	100	25	10
1.6	160	40	16
2.4	240	60	24
4,0	—	—	40
6,0	—	—	60

The third set of experiment were carried out at two temperatures (10 °C and 50 °C) with two pulse repetition frequencies (50 pps and 500 pps) under neutral condition. The temperature, which differed from room temperature, was kept constant with a T4600 Lauda process thermostat. All experiments were carried out under ambient pressure with 50 mg/L initial prepared concentration of sulfamethizole. A list of all experiments with operating parameters is shown in Table 1. With the exception of experiments without power supply, all experiments were repeated 4 times in order to improve accuracy and make sure that the experiments are reproducible. The standard deviation did not exceed 0.05 for all experiments.

### Data Availability

The datasets generated during the current study are available from the corresponding author on reasonable request.

## Results and Discussion

As mentioned earlier, three sets of experiments were carried out (see section 2, Table 1). In this section the three sets are considered separately and then a summary of the main findings is given.

### First set of experiments

The experiments without power supply showed that the sulfamethizole is stable and does not degrade itself. No changes in sulfamethizole concentration were detected. Neither pH nor temperature had any effect on oxidation without power supply. The experiments were carried under ambient pressure and at room temperature (20 °С).

### Second set of experiments

**Figure 2:**
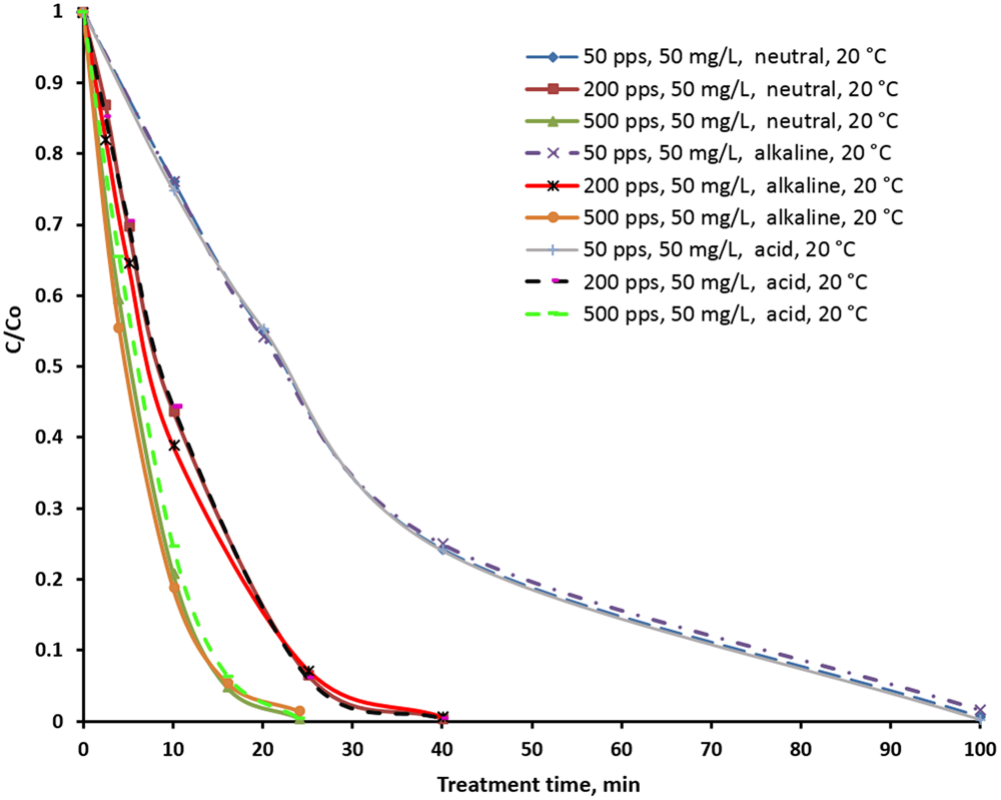
Relationship between sulfamethizole relative concentration (C/C_0_) and treatment time at different pulse frequencies (50 pps, 200 pps, 500 pps) and initial pH. Temperature of treated solution is 20 °C.

To investigate the effect of temperature, the general behavior of sulfamethizole in a cold plasma field should be studied. This was the main goal of the second set of experiments. Figure 2 shows the oxidation curves of sulfamethizole with the 4.5 L/min flow rate of circulating aqueous solution. It is possible to conclude that sulfamethizole is easily oxidized by PCD. The highest degradation was observed at the beginning of the PCD treatment followed by a deceleration. The concentration of sulfamethizole decreases below the measurement limit after 24 min, 40 and 100 min of treatment time at 500 pps, 200 pps, and 50 pps respectively. As can be seen from the figure, initial pH has no significant effect on the oxidation. At 50 pps, the three oxidation curves merge into one. At higher frequencies, a slight difference in the behavior of the oxidation curves can be observed in the case of alkaline media. It seems that high pH is a little bit more preferable for oxidation, but in general, the effect of pH is insignificant. It should be noted that throughout the treatment process the temperature remained constant (around 20 °C) regardless of treatment time or energy supplied. The experiments with 8 L/min of circulating aqueous solution gave similar results, indicating that the flow rate has no effect on the process.

The unknown quantity of OH-radicals and ozone, as well as lack of knowledge about their individual contribution to the reaction may pose a considerable challenge for calculation of the reaction kinetics. Taking into account that the water flow rate has no effect on the process and assuming that contact surface remains constant, it is possible to conclude that there are constant amounts of oxidants available at any moment in the plasma zone. Therefore, the reaction rate constant can be calculated by assuming that the combined effect of the oxidants results in a second order reaction rate (first order relative to the sulfamethizole and first order relative to oxidants). Power delivered to the volume of the plasma zone can be used for characterization of the total amount of oxidants involved the process:

6 $$dC/dt={k}_{2}CP/{V}_{pl}$$

where *k*_2_ is the second order reaction rate constant (in m^3^ J^−1^), *C* is the concentration of the sulfamethizole (in mg/L), *P* is the pulse power delivered to the reactor (in W), and *V*_*pl*_ is the plasma zone volume (in m^3^).

The *V* value depends on the reactor design, in our case *V*_*pl*_ = 0.00714 m^3^, and the P value depends on the pulse repetition frequency (see section 2). For each experiment, *P/V*_*pl*_ is a constant, and therefore it is possible to rewrite Eq. (6) in the following way:

7 $$dC/dt={k}_{1}C$$

Equation 7 is a first order reaction, where *k*_1_ (in min^−1^) is a pseudo-first order reaction rate constant:

8 $${k}_{1}={k}_{2}P/{V}_{pl}$$

In the case of a first order reaction, the function of the change in concentration with the treatment time should behave by exponential law. An approximation of the experimental data was made using the MatLab software package. Oxidation curves for all experiments are functions of exponential type with a coefficient of determination not less than 0.99, which indicates a first order reaction. The *k*_1_ value, calculated by MatLab, and *k*_2_, calculated from Eq. 8, are given in Table 1. As can be seen, the pseudo-first order reaction rate constant grows with increase in pulse repetition frequency and does not depend on pH. In the case of the calculated *k*_2_ values, by contrast, decreasing frequency leads to a reduction of the second order reaction rate constant. The obtained kinetics results are comparable with other studies. For example, Chamberlain and Adams^33^ utilized chlorine and monochloramine for sulfamethizole oxidation. In their study, the reported values of a pseudo-first order reaction rate constant (0.015 min^−1^, 0.021 min^−1^ and 0.006 min^−1^) are lower than the results found herein.

The experimental results in this work showed that after 1 kWh/m^3^ delivered energy the great part of sulfamethizole was oxidized. The dependence of sulfamethizole concentration on delivered energy is presented in Supplementary Figure S1. It can be observed that the lowest frequency leads to the fastest degradation rate. With the lowest frequency (50 pps), sulfamethizole seems to have been completely oxidized at around 1 kWh/m^3^, whereas operating with 500 pps more than 2 kWh/m^3^ is required to reduce the sulfamethizole concentration to the detection limit.

Energy efficiency is calculated by Equation (5). The most common ways of calculating energy efficiency are half-life energy efficiency (*ε*_1*/2*_) at treatment time equal to a 50% reduction in the target compound (*t*_1*/*2_), and energy efficiency when compound removal approximates 100%, which can be termed the final energy efficiency.

Knowing *k*_*1*_ values, it is possible to calculate *t*_*1/2*_ by the following equation:

9 $${t}_{1/2}=ln2/{k}_{1}$$

Substituting *t*_*1/2*_ in Eq. (5) gives the *ε*_*1/2*_ value.

**Figure 3:**
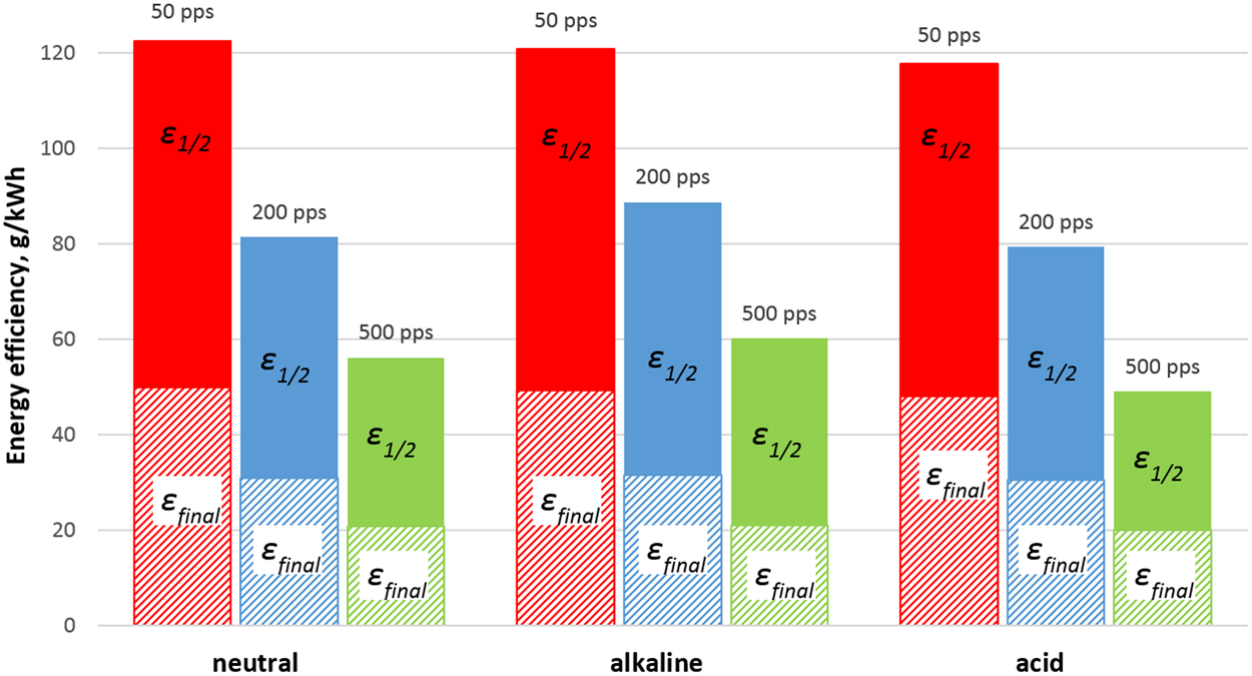
Energy efficiency of sulfamethizole degradation at different pulse frequencies (50 pps, 200 pps, 500 pps) and initial pH. Hatched area – final energy efficiency (*ε*_*final*_), solid area – half-life energy efficiency (*ε*_*1/2*_).

As mentioned earlier, in this study the sulfamethizole seems to have been completely oxidized after 24 min, 40 and 100 min of treatment time at 500 pps, 200 pps, and 50 pps, respectively. The final energy efficiency was calculated for these times for maximum compound removal (R = 9%). The calculated values of *ε*_*1/2*_, and *ε*_*final*_ are given in Table 1 and shown for visual clarity also in Fig. 3. As can be seen, the maximum half-life energy efficiency, around 120 g/kWh, was achieved at 50 pps, which is 1.5 and 2.2 times higher than with the experiments at 200 pps, and 500 pps respectively. A similar trend persists in the case of final energy efficiency. It should be noted that pH has insignificant effect on both efficiencies. The better result, from the energy efficiency point of view, with the low pulse frequency can be explained by the greater contribution of ozone in the oxidation process. Hydroxyl radicals and ozone react with target compounds directly in the gas-liquid interface. Ozone has lower oxidation potential than OH-radicals, and it reacts with target compound more slowly; furthermore, when dissolved in water ozone may also decompose via formation of OH-radicals. Such formation of OH-radicals can be considered as secondary formation. Dissolving of ozone and secondary formation of OH-radicals take time. In the case of experiments with low pulse frequency, the treatment time to reach the same value of delivered energy increases compared with the high pulse frequency experiments (see Table 2). Consequently, ozone has more time to accumulate during the pauses between the pulses, and more time to dissolve and for the reaction. The results for the impact of ozone on the oxidation process are consistent with earlier gas-phase PCD studies^34,35^.

Oxidation of sulfamethizole leads to the formation of several transformation products. Qualitative analysis focused on identification of organic by-products only. Except sulfamethizole itself, the 4 identified organic products have highest peak at the half-life oxidation time (see Table 3). Some other compounds, including organic acids, were also detected and are given in Supplementary Figure S3. The presence of organic acids explain the decreasing of pH during PCD treatment. This is especially noticeable with neutral initial pH^36^. As 2-amino-5-methyl-1,3,4-thiadiazole was also observed in the samples, it is reasonable to assume that possible reaction pathways start with preliminary hydroxylation of sulfamethizole with subsequent sulfonamide bond breaking. Similar pathways were suggested by Klauson *et al*.^25^. At the end of the treatment time, when sulfamethizole compound removal reached a maximum (R = 99%) none of the mentioned intermediates were detected at the resolution of the analysis method used.

**Table 3:** Oxidation by-products with the highest peak at the half-life oxidation time.

Compound	Structure	Identified with	note
Sulfamethizole		Ion trap, positive Ion trap, negative QToF, positive QToF, negative	4 fragments 3 fragments error 3,7 ppm error 3,7 ppm
OH-Sulfamethizole		Ion trap, positive Ion trap, negative	3 fragments 3 fragments
3 OH-Sulfamethizole		Ion trap, negative	2 fragments
4 OH-Sulfamethizole		Ion trap, negative QTOF negative	2 fragments error 6,1 ppm
Carboxy- Sulfamethizole		Ion trap, negative QToF, negative	2 fragments error 9,9 ppm

Based on the second set of experiments, it is possible to present a number of interim findings. pH and circulating aqueous flow rate have no significant effect on the oxidation process either in terms of energy efficiency or reaction kinetics. Treatment at low frequency is preferable from the energy efficiency point of view. The second reaction rate constant decreases with increasing pulse repetition frequency. The ozone as an oxidant starts to play a more significant role in the oxidation process at low pulse frequency. None of the transformation products detected at the time of 50% sulfamethizole compound removal were identified during the final period of the treatment process.

### Third set of experiment

As the oxidation process is not dependent on pH and flow rate, experiments with temperature variation were carried out only in neutral media and only with 4.5 L/min flow rate. Pulse frequency of 50 pps and 500 pps were used in these experiments. Three temperatures were tested – room temperature (approx. 20 °C), 10 °C and 50 °C.

**Figure 4:**
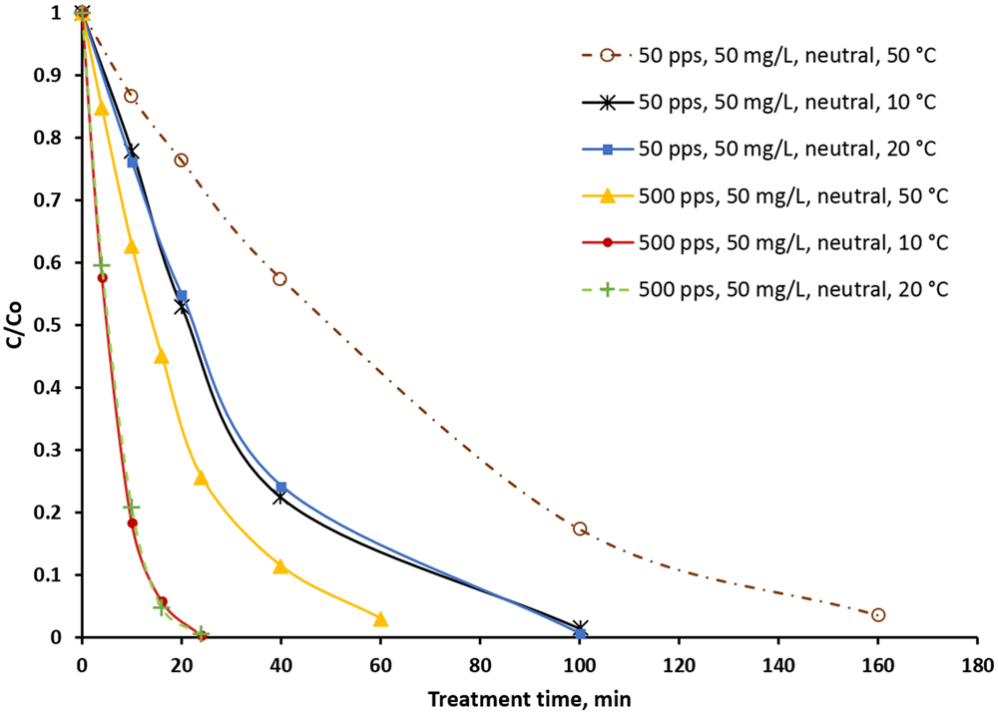
Sulfamethizole relative concentration (C/C_0_) vs treatment time at different temperature (10 °C, 20 °C, and 50 °C) and pulse frequencies (50 pps and 500 pps). Initial pH is neutral.

**Figure 5:**
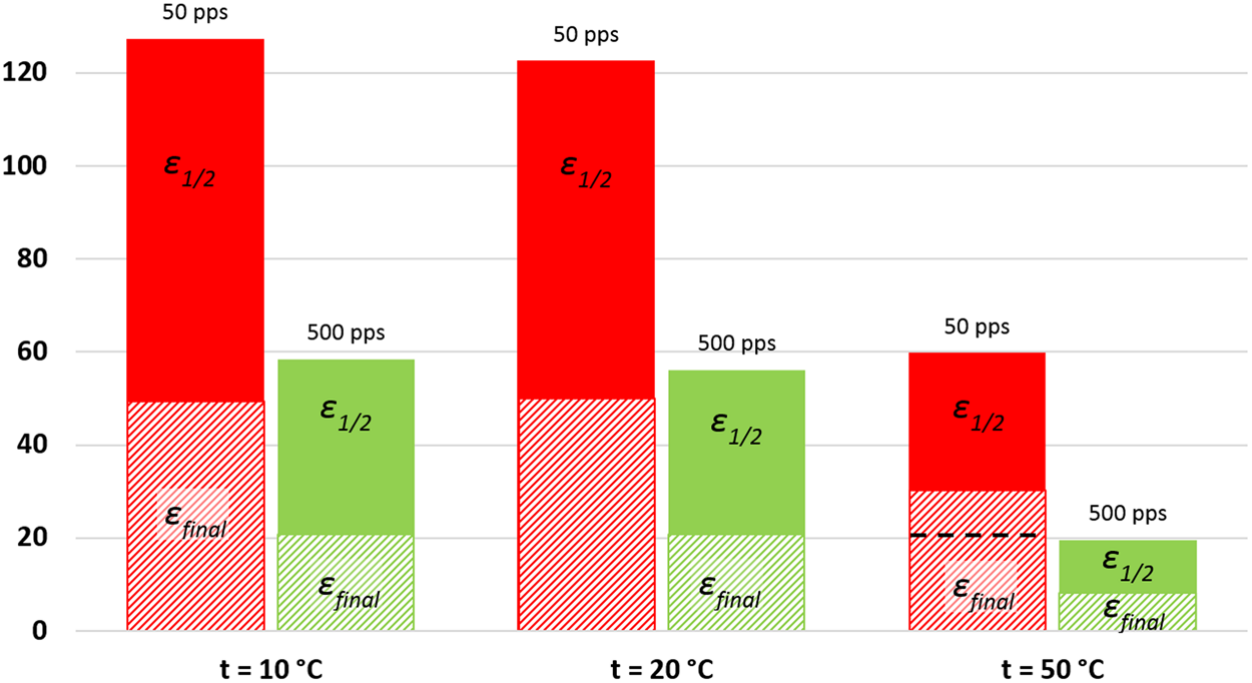
Energy efficiency of sulfamethizole degradation at different pulse frequencies (50 pps, 500 pps) and temperature (10 °C, 20 °C, and 50 °C). Initial pH is neutral. Hatched area – final energy efficiency (*ε*_*final*_), solid area – half-life energy efficiency (*ε*_*1/2*_), dash line – *ε*1/2 value after approximation by Matlab).

Figure 4 shows the oxidation curves of sulfamethizole at different temperatures. As can been seen, there is no difference between treatments at 10 °C and room temperature, whereas a temperature of 50 °C leads to significant deceleration of the oxidation process. 24 and 100 minutes treatment time at 500 pps and 50 pps respectively were sufficient for almost complete removal of sulfamethizole, but at a temperature of 50 °C and with the same frequencies, removal takes 60 and 160 minutes. Nevertheless, the dependency of concentration changes on treatment time remains exponential. Reaction order, reaction constant, half-life oxidation time, and energy efficiency were determined in the same way as described in section 3.2 (see Table 1). All values in Table 1 were calculated based on experimental data. Energy efficiency at different temperatures is shown in Fig. 5.

At elevated temperature of 50 °C, it is possible to observe a decrease in the *k*_*1*_, *k*_*2*_, *ε*_*1/2*_, values and increase in the *t*_*1/2*_ value of 2 and 2.9 times at 50 pps and 500 pps respectively. Half-life energy efficiency at 50 °C and 50 pps becomes almost equal to the half-life energy efficiency at 20 °C (10 °C) and 500 pps. With the temperature of 50 °C, the final energy efficiency decreased 1.7 times at 50 pps and 2.5 times at 500 pps (see Fig. 5). It is worth mentioning that unlike the second set experiments, when *ε*_*1/2*_ was calculated for R = 0.99 with increased temperature, the removal rate was 0.97 in the calculation of final energy efficiency. The curve (relative concentration vs delivered energy) at 50 °C and 50 pps almost merges with the curves at 20 °C and 10 °C and 500 pps (see Supplementary Figure S2). It is reasonable to assume that if the process is prolonged at 50 °C and 50 pps until compound removal reaches 0.99, the final energy efficiency will also become equal to the final energy efficiency at 20 °C (10 °C) and 500 pps. An approximation made by Matlab software lends support to this assumption (See Table 1).

The drop in oxidation process speed and energy efficiency at elevated temperature can be explained by the decrease in ozone solubility with temperature increase. Moreover, decomposition of ozone increases at higher temperature. As mentioned earlier, ozone can react with the target compound directly on the border of liquid-gas interface or in the bulk. Dissolved ozone also reacts with compounds in the solution by formation of OH radicals. The lower ozone solubility in the aqueous solution at higher temperature leads to decrease in the secondary formation of OH radicals, which in turn slows down the OH radical-induced oxidation; and, secondly, more ozone becomes available for direct reaction on the border of liquid-gas interface. However, gaseous ozone decomposes according to the equation 2О_3_=3О_2_. Elevation of the temperature accelerates the ozone decomposition and the balance shifts towards oxygen formation. Oxygen, in turn, has lower oxidation potential compared to ozone. Therefore, the role of ozone in such a case is small. It should be noted here that when discussing the temperature, the temperature of the treated solution is meant. The temperature in the plasma zone consisting of a continuous gas phase and dispersed liquid droplets is slightly different.

The results in this set of experiments are slightly inconsistent with those of the second set of experiments. It was concluded based on the second set of experiments that ozone starts to play more significant role in the oxidation process at lower pulse frequencies. Consequently, excluding ozone from the process, it was reasonable to assume that the oxidation reaction will slow to a greater degree at lower frequency than higher frequency, but the results evidence the opposite. It is possible to explain this in the following way: already at 40 °C the ozone solubility is about zero, and thus it can be considered that there is no ozone in the bulk solution. However, there is still gaseous ozone in the plasma zone, and oxygen formed after ozone decomposition and atomic oxygen are also present in the gas phase. Such oxidants require longer reaction time, as they are less reactive towards the target compound comparing with OH radicals, and treatment at lower frequency extends the time available for the reaction. Therefore, even with high temperature, a low frequency is preferable for sulfamethizole oxidation from the energy efficiency point of view. Based on this hypothesis, a reduction in temperature should be followed by intensification of the oxidation process. However, the same results were observed with 10 °C and room temperature. It appears that the potential increase in energy efficiency at lower temperature as a consequence of increased ozone reactivity is compensated by the increase in the reaction speed with a factor 2 or 3 per 10 °C according to the Van ‘t Hoff equation. That is why, change in temperature of water between 10 °C and room temperature does not affect the outcome of the sulfamethizole oxidation process. Such temperature range corresponds to the average and maximum water surface temperature in Finland. Thus, PCD technology can be applied for water purification or disinfection just after potable abstraction from water bodies. From the point of view of energy efficiency, the results of this study show that PCD technology is an effective method for sulfamethizole removal. For comparison, Klauson *et al*.^25^, who implemented aqueous photocatalytic oxidation, managed to obtain a maximum 21 g/kWh after removal of 25% of target compound. With PCD treatment, the best result gives almost 6 times better efficiency, which is an indication of the considerable potential benefits from PCD utilization.

## Conclusion

The plasma field in the PCD reactor promotes fast and effective oxidation of sulfamethizole. By replacing oxidants concentration with power delivered to the plasma zone, the oxidation reaction could be expressed by a second order reaction and then well approximated as a pseudo-first order reaction.

The parent compound and its aromatic by-products seemed to be fully degraded after relatively short treatment time and with low energy consumption. Neither pH nor water recirculation flow rate had any significant effect on the process. Treatment temperature of 50 °C dramatically decreased energy efficiency and slowed down the process compared to room temperature. No differences in oxidation efficiencies were observed between the results obtained at room temperature and at 10 °C. Treatment at low pulse frequency is preferable from the energy efficiency point at any temperature within the studied framework. In the case of low frequency treatment, ozone has more time to react with target compound and makes the most significant contribution to the oxidation process at low frequency. However, temperature increasing leads to decreasing of ozone contribution due to reducing its solubility in water.

## Electronic supplementary material

Supplementary Information
