# Supplementary material for: Behaviour of aqueous sulfamethizole solution and temperature effects in cold plasma oxidation treatment
Source: Sci Rep. 2018 Jun 7;8:8734. doi: 10.1038/s41598-018-27061-5 (PMC5992216; doi:10.1038/s41598-018-27061-5)
Supplement: Supplementary file 1 — Supplementary Information [file 41598_2018_27061_MOESM1_ESM.docx]

Behaviour of aqueous sulfamethizole solution and temperature effects in cold plasma oxidation treatment

Alexander Sokolov^1^, Marjatta Louhi-Kultanen^1,2^

Supplementary Information

Figure S1 Sulfamethizole relative concentration vs delivered energy

Figure S2 Sulfamethizole relative concentration vs delivered energy at different temperature


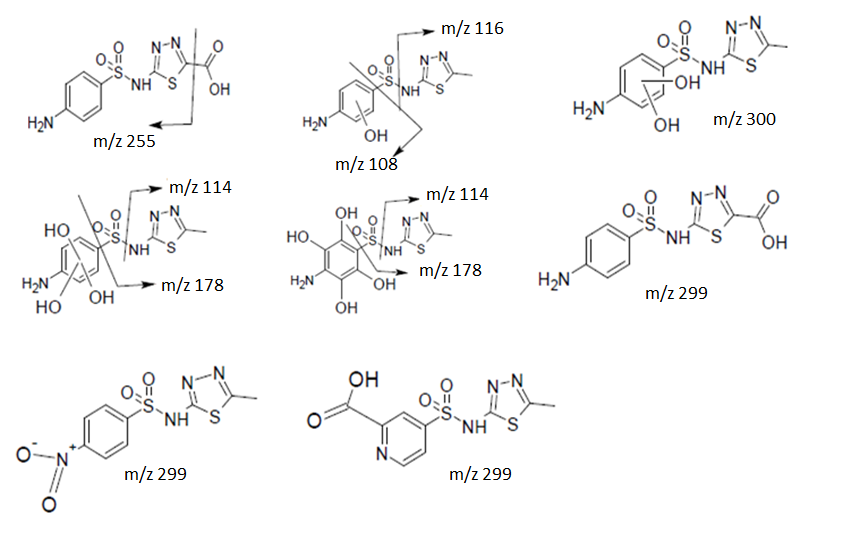


Figure S3 Oxidation by-products with m/z values
